# Ultrasound-Guided Nerve Blocks Have the Potential to Reduce Racial and Ethnic Disparities in Emergency Department Pain Management 

**DOI:** 10.24908/pocus.v9i2.17510

**Published:** 2024-11-29

**Authors:** Kendall Lavin-Parsons, Zachary W Binder

**Affiliations:** 1 UMass Chan Medical School, University of Massachusetts Worcester, MA USA

**Keywords:** Ultrasound-Guided Nerve Block, Nerve Block, Regional Anesthesia, Point-of-Care Ultrasound Guided Regional Anesthesia, Equity

## Introduction

Injuries are a common presenting complaint in the emergency department 1. Pain from injuries has traditionally been treated with opioids 2. Extensive research exists describing the potential adverse effects of systemic opioids including bradypnea, hypotension, and central nervous system depression. Further complicating the use of opioids is that they are difficult to titrate and can lead to dependence and addiction 3. Ultrasound-guided nerve blocks (UGNB) are an effective alternative to opioids as they safely and effectively control pain while mitigating adverse systemic effects 4. There is emerging evidence supporting the use of nerve blocks in treating long bone fractures, rib fractures, extremity wounds, joint dislocations, retained foreign bodies, and many other painful conditions 5, 6. We propose an additional benefit to the use of this modality; UGNB can lead to more equitable care. 

Racial and ethnic biases are unfortunately pervasive in medicine. While a problem across all of healthcare, racial and ethnic biases are perhaps most disturbing in pediatrics. In 2020, the Pediatric Emergency Care Applied Research Network (PECARN) published an eye-opening study demonstrating that minority children who presented to the hospital with fractures were less likely to receive opiate pain medications than their White Non-Hispanic counterparts and were less likely to achieve optimal pain reduction 7. It is these findings that sparked our interest in the use of UGNB as a tool for change.

### Causes of Systemic Bias in the Emergency Department

Medical provider implicit biases have been implicated as the leading cause of racial and ethnic disparities in pain control 8. Several factors help give rise to this problem. It has been shown that providers perceive less pain in African Americans, compared to other races, and subsequently document and base course of treatment on such perceptions 9. Another factor is the adultification of African American children. Adultification is the idea that adults perceive Black children as older than they are. This concept has broad implications throughout society, particularly in medicine, and can lead to the undertreatment of pain 10. Lastly, there is an increasing lack of empathy for families of lower socioeconomic status, who are disproportionately minorities, forced to utilize an already overcrowded emergency department for their primary care. All of these factors are exacerbated in the Emergency Department (ED). The inherent speed and stress associated with the ED diminish the conscious brain’s involvement in decision-making, thus limiting a provider’s ability to combat their own implicit biases 11.

### Opioid Crisis

It is important to recognize the role of the opiate crisis in this issue. There was a steady rise in opioid prescriptions beginning in the 1990s and continuing through the early 2000s, resulting in a rapid increase in overdose-related deaths. In 2011 the CDC declared the “Opioid Epidemic.” This crisis disproportionately affected the poor and marginalized within our society, who were inordinately people of color. As a result, an exaggerated association developed between people of color and the dangers of opioids 12.

### Current Efforts in Place to Tackle Racial and Ethnic Bias

The AAMC and ACGME have begun to recognize the role of provider bias in the treatment of patients. To combat this, they have started to modify their training and competency requirements 13. In a survey distributed to pediatric residents, implicit bias was identified as requiring further education. Fortunately, most residents expressed interest in receiving training aimed at reducing their own biases 14. Initiatives aimed at addressing provider bias are often program-specific and limited to discussion-based longitudinal courses. A study that evaluated pre- and post-survey responses as part of one such course found only modest effects 15. 

### Ultrasound-Guided Nerve Blocks Have the Potential to Reduce Inequity

The incorporation of UGNB as a standard of care treatment for injuries would reduce inequities in pain management. The efficacy of a UGNB is not impacted by a patient’s age, gender, race, or ethnicity or on the caregiver’s perception of their patient’s pain. Even if a provider’s perception of a patient’s pain is biased, the nerve block will still reduce the patient’s pain. Additionally, because UGNB can last up to 18 hours 16, they reduce the reliance on pain reassessment and medication titration. 

## Conclusions

Provider bias has been implicated as the number one cause of racial and ethnic disparities in pain management. The declaration of the “Opioid Epidemic”, while a necessary wake-up call to physicians, has had a compounding effect on this problem. Factors such as a lack of cultural competency surrounding pain reporting, ED overcrowding due to improper utilization of this level of care, the speed and stress of working in the ED, and the adultification of African American children, all contribute to this inequity. UGNBs do not address the underlying causes of these disparities, however, when ubiquitously implemented could improve equity. We advocate for improved longitudinal anti-bias education, combined with the training of UGNBs to improve racial and ethnic disparities in pain management.
